# Analysis of Genomic Characteristics of SARS-CoV-2 in Italy, 29 January to 27 March 2020

**DOI:** 10.3390/v14030472

**Published:** 2022-02-25

**Authors:** Alessandra Lo Presti, Angela Di Martino, Giovanni Faggioni, Francesco Giordani, Silvia Fillo, Anna Anselmo, Vanessa Vera Fain, Antonella Fortunato, Giancarlo Petralito, Filippo Molinari, Stefano Palomba, Riccardo De Santis, Stefano Fiore, Concetta Fabiani, Giuseppina Di Mario, Marzia Facchini, Laura Calzoletti, Florigio Lista, Giovanni Rezza, Paola Stefanelli

**Affiliations:** 1 Department of Infectious Diseases, Istituto Superiore di Sanità, 00161 Rome, Italy; angela.dimartino@iss.it (A.D.M.); stefano.fiore@iss.it (S.F.); concetta.fabiani@iss.it (C.F.); giuseppina.dimario@iss.it (G.D.M.); marzia.facchini@iss.it (M.F.); laura.calzoletti@iss.it (L.C.); paola.stefanelli@iss.it (P.S.); 2 Scientific Department, Army Medical Center, 00184 Rome, Italy; giovanni.faggioni@gmail.com (G.F.); franc.giordani@gmail.com (F.G.); silviafillo@gmail.com (S.F.); annanselm@gmail.com (A.A.); fainvanessavera@gmail.com (V.V.F.); antonellafortunato75@gmail.com (A.F.); giancapetra@gmail.com (G.P.); molinarifilippo@hotmail.com (F.M.); riccardo.desantis@gmail.com (R.D.S.); romano.lista@gmail.com (F.L.); 3 Department of Science, University of Rome “Roma Tre”, 00146 Rome, Italy; 4 General Directorate of Military Medical Services-Medical Situation Awareness Branch, 00184 Rome, Italy; stefano.palomba@gmail.com; 5 Health Prevention Directorate, Ministry of Health, 00144 Rome, Italy; g.rezza@sanita.it

**Keywords:** SARS-CoV-2 evolution, genomics, gene flows, dated phylogeny

## Abstract

We performed next-generation sequencing (NGS), phylogenetic analysis, gene flows, and *N*- and *O*-glycosylation prediction on SARS-CoV-2 genomes collected from lab-confirmed cases from different Italian regions. To this end, a total of 111 SARS-CoV-2 genomes collected in Italy between 29 January and 27 March 2020 were investigated. The majority of the genomes belonged to lineage B.1, with some descendant lineages. The gene flow analysis showed that the spread occurred mainly from the north to the center and to the south of Italy, as confirmed by epidemiological data. The mean evolutionary rate estimated here was 8.731 × 10^−4^ (95% highest posterior density, HPD intervals 5.809 × 10^−4^ to 1.19 × 10^−3^), in line with values reported by other authors. The dated phylogeny suggested that SARS-CoV-2 lineage B.1 probably entered Italy between the end of January and early February 2020. Continuous molecular surveillance is needed to trace virus circulation and evolution.

## 1. Introduction

Human coronaviruses (CoV) are enveloped positive-stranded RNA viruses belonging to the order *Nidovirales*, mostly responsible for upper respiratory and digestive tract infections [1].

An outbreak of a febrile respiratory illness due to the newly discovered coronavirus (officially named by the World Health Organization as SARS-CoV-2 (COVID-19)) occurred in mid-December 2019, in the city of Wuhan, Hubei province (China). The virus spread across most countries on all continents, causing a pandemic event [2,3,4].

The first patients with confirmed COVID-19 diagnosed in Italy were two Chinese tourists hospitalized in Rome [5]. Moreover, a case was identified on 20 February 2020 in Lombardy Region (Codogno), Northern Italy [6]. The virus spread through the country very rapidly, causing the first epidemic wave, which was characterized by a high number of cases and deaths [7].

On 9 March 2020, considering the excessive number of patients admitted to the intensive care unit, a lockdown was declared for the entire country as a stringent measure for the containment of SARS-CoV-2 circulation. The lockdown regarded restrictions on the mobility of the population except for necessity, work, and health circumstances, respecting defined curfew times, in addition to social distancing and closure of all non-essential services. During that period, the majority of cases occurred in northern parts of Italy.

The availability of whole-genome sequences collected over time can be useful for molecular surveillance of the epidemic and for evaluation and planning of effective control strategies. To understand virus evolution, we performed a next-generation sequencing (NGS) analysis on SARS-CoV-2 genomes collected from lab-confirmed samples during the early stage of the COVID-19 pandemic. Phylogenetic analysis was carried out to characterize SARS-CoV-2 genomes and to identify the dynamics of local spread. A broad comparison of SARS-CoV-2 isolates with different geographic origin and dating from the early phase of COVID-19 outbreaks was performed to estimate the rate of evolution and the dissemination pathways of SARS-CoV-2.

Previous studies [8,9,10] have provided a genomic snapshot of the viral lineages circulating in some Italian regions, the timescale and phylodynamics of the Italian SARS-CoV-2 epidemic, but analyzing sequences from north and central Italy, predominantly. Another paper aimed to track circulation between a major number of Italian regions [11].

None of the above-reported studies have coupled the “classic” phylogenetic analysis with the “gene flow” alternative approach to estimate the significant migration events, as well as simultaneously analyzing the genetic diversity and glycosylation sites’ prediction on SARS-CoV-2 sequences collected from a large number of Italian regions.

Specifically, the aims of this study were: (i) to investigate the gene flows among different Italian areas (north, center, south), giving a clear picture of the predominant strains causing disease at that time, (ii) to investigate the genetic diversity and the timescale phylogeny of SARS-CoV-2 strains collected from different Italian regions during the early phase of the epidemic, and (iii) finally, to investigate the *N*- and *O*-glycosylation sites’ prediction on the *spike protein* on a large dataset with a geographical representation.

## 2. Materials and Methods

### 2.1. Maximum Likelihood Phylogenetic Analysis

The Italian dataset consisted of 111 genomes, of which 58 are sequenced strains collected at the Istituto Superiore di Sanità, Rome, Italy, and from the Italian Scientific Department of the Army Medical Center (46 of them newly sequenced and 12 previously submitted to GISAID), while 53 are genomes of Italian origin collected from other institutes/hospitals and available in the GISAID database [12]. The samples were selected according to their viral titer estimated by the resulting real-time PCR cycle threshold (Ct) value (from 16 to 25 cycles). Sequences containing >0.1% of ambiguous nucleotides (N) were detected with an ad hoc script and removed from the dataset. Among the samples obtained during the study period (from 29 January until 27 March 2020), only six were excluded because of a high Ct value (30–35), and two for their high content of ambiguous nucleotides. The viral RNA was extracted using the QIAMP VIRAL RNA Mini Kit or RNeasy Mini Kit (Qiagen, Hilden, Germany) and retro-transcribed using the SuperScript III Reverse Transcriptase kit (Invitrogen, Carisbad, CA, USA). Double-stranded DNAs were subsequently obtained by Klenow enzyme (Roche, Basel, Switzerland) according to the manufacturer’s instructions. The Nextera XT kit (Illumina, San Diego, CA, USA) was used for library preparations and whole-genome sequencing was performed using the Illumina Miseq Reagent V2 (2 × 150 cycles) or the Illumina NextSeq 500 High Output Kit V 2.5 (2 × 150) (Illumina, San Diego, CA, USA) on the Illumina MiSeq or NextSeq 500 instruments, respectively. The reads were trimmed for quality (qscore ≥ 20) and minimum length (=100) using the BBDuk trimmer. High-quality reads were assembled by mapping to the reference genome from Wuhan, China (GenBank an. NC_045512.2), with the bowtie2 mapping algorithm integrated in Geneious Prime software (www.geneious.com accessed on 18 June 2020).

The collection dates of the Italian dataset ranged from 29 January until 27 March 2020. The sequence alignment was performed by using MAFFT v7 [13] under the Galaxy platform [8] and manually edited by using BioEdit v. 7.2.6.1 [14]. The best-fitting substitution model was estimated by means of J Modeltest 2 [15].

To explore the lineages of the 58 SARS-CoV-2 Italian genomes, the “Pangolin COVID-19 Lineage Assigner” [16] (version 3.1.14, lineage version 13 October 2021) was adopted in order to assign the lineages, on the basis of the methodology described by Rambaut [17].

The phylogenetic tree of this dataset was constructed by the maximum likelihood method by means of the IQ-TREE software version 1.6.11 [18,19] under the general time-reversible nucleotide substitution model with a proportion of invariant sites (GTR + I + G), which was previously inferred in jModelTest. Statistical support has been inferred by both the SH-like aLRT and the bootstrap analysis (1000 replicates). The tree was visualized by means of FigTree software v.1.4.4 [20].

### 2.2. Mutations and Glycosylation Pattern

The mutations were identified by investigation of the sequence alignments. The glycosylation pattern of the SARS-CoV-2 *surface* glycoprotein was analyzed by means of the N-GlycoSite program [21,22] to characterize and predict potential N-linked glycosylation sites. Furthermore, we aimed to perform the prediction of the potential *O*-glycosylation sites in the SARS-CoV-2 surface glycoprotein by using Net O Glyc v. 4.0.0.13 software [23].

### 2.3. SARS-CoV-2 Gene Flows, Evolutionary Rate Estimate, and Time-Scaled Phylogeny among Italian Regions

The Mac Clade program version 4 [24,25,26] was used to test gene out/inflow in Italy among SARS-CoV-2-infected subjects from different areas (north, center and south) and Italian regions by using a modified version of the Slatkin and Maddison test [26]. The maximum likelihood tree, previously reconstructed, was imported into Mac Clade and used as the starting tree for the gene flow analysis. A one-character data matrix was obtained from the dataset by assigning to each taxon in the tree a one-letter code indicating its own sampling location, according to three areas of Italy (north, center and south) and to different Italian regions.

The putative origin of each ancestral sequence (i.e., internal node) in the tree was inferred by finding the most parsimonious reconstruction (MPR) of the ancestral character. The final tree length, that is the number of observed gene flow events in the genealogy, can easily be computed and compared to the tree-length distribution of 10,000 trees obtained by random joining–splitting (null distribution). Observed genealogies significantly shorter than random trees indicated the presence of subdivided populations. Specific migrations among different areas and regions were traced with the state changes and stasis tool (Mac Clade), which counts the number of changes in a tree for each pairwise state, as previously described [26]. When multiple MPRs were present, the algorithm calculated the average migration count over all possible MPRs for each pair. The resulting pairwise migration matrix was normalized to obtain the percentage of observed migration to/from different areas or regions of Italy in the tree. Only statistically supported gene flow events were reported. The null hypothesis of panmixia (i.e., no population subdivision or complete intermixing of sequences from different geographic areas) was rejected by the randomization test (*p* < 0.0001).

The Italian dataset was further investigated to estimate the mean evolutionary rate and the time-scaled phylogenetic tree. All the Italian sequences (*n* = 111) together with four genomes from Wuhan, collected during the first phase of the epidemic (EPI_ISL_ 402119, EPI_ISL_ 402121, EPI_ISL_402123, and EPI_ISL_402124), one German (EPI_ISL_406862), and three Chinese from Shanghai known to be ancestral to the B.1 clade (EPI_ISL_416327, EPI_ISL_416334, EPI_ISL_416386), were added in this analysis for dating the epidemic, as previously reported [8].

A root-to-tip regression analysis was performed using TempEst in order to investigate the temporal signal of the dataset [27]. The Bayesian time-scaled tree and the mean evolutionary rate were co-estimated with the Beast program, v. 1.10.4 [28], by using the GTR + G + I model of nucleotide substitution, as previously estimated. As coalescent priors, different demographic models (a constant population size, exponential growth, and the Bayesian skyline plot (BSP) and strict vs. relaxed molecular clock models were tested by means of path sampling (PS) and stepping stone (SS) sampling [29]. The (log) Bayes factors between two competing and alternative models, M_0_ and M_1_, were compared in order to select the models (clock and demographic) that best-fit the data. M_0_ typically represents the null hypothesis, and the evidence in favor of or against a null hypothesis was evaluated.

Kass and Raftery [30] introduced different gradations to assess the log Bayes factor as evidence against M_0_. A value between 0 and 1 is not worth more than a bare mention, whereas a value between 1 and 3 is considered as positive evidence against M_0_. Values larger than 3 and 5 are considered to respectively give strong and very strong evidence against M_0_ [30,31].

The evolutionary rate prior was set as a normal distribution, as previously described [32,33,34]. A tree search was carried out running a Markov chain Monte Carlo (MCMC) for 100 million generations (initial burn in of 10%), sampling every 10,000th generation. Convergence of the MCMC was assessed by calculating the ESS for each parameter. Only values of ESS > 200 were considered significant. The maximum clade credibility tree was obtained from the trees’ posterior distributions with the Tree-Annotator software v 1.10.4, and statistical support for specific monophyletic clades was assessed by calculating the posterior probability.

The tree was visualized by means of FigTree software v.1.4.4 [20].

### 2.4. Ethical Approval

The study was approved by the Ethical Committee of the ISS (Prot. PRE BIO CE No. 26259—29 July 2020).

## 3. Results

### 3.1. Phylogenetic Assignment through Pangolin COVID-19 Lineage Assigner and Maximum Likelihood Phylogenetic Analysis

**Figure 1:**
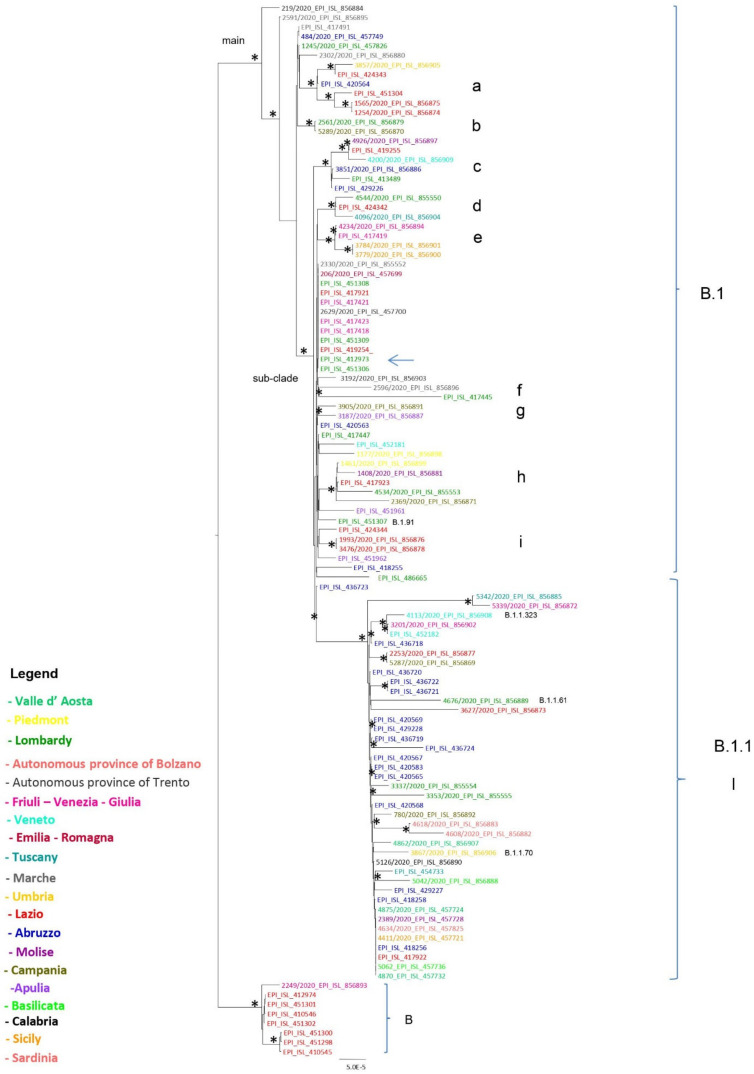
Maximum likelihood phylogenetic analysis of 58 SARS-CoV-2 Italian genomes from the Istituto Superiore di Sanità (Rome, Italy) and from the Italian Scientific Department of the Army Medical Center, plus 53 complete Italian genomes downloaded from GISAID (collected from other Institutes). The tree was rooted according to the cluster highlighted at the bottom of the figure, corresponding to SARS-CoV-2 lineage B sequences, as they are divergent from the rest of the taxa (B.1 and descendant lineages) and to give directionality to the tree. Branch lengths were estimated with the best-fitting nucleotide substitution model according to a hierarchical likelihood ratio test. The scale bar at the bottom represents nucleotide substitutions per site. An asterisk along a branch represents significant statistical support for the clusters subtending that branch (bootstrap support and aLRT > 80%). The main clades and clusters are highlighted. The colors of the tips represent strains from different Italian regions (Abruzzo, blue; Lazio, red; Lombardy, green; Friuli-Venezia Giulia, pink-fuchsia; Marche, grey; Veneto, light blue; Molise, violet; Sicily, ocra yellow; Sardinia, pink flesh; Campania, dark green; autonomous province (AP) of Trento, dark grey; Umbria, intermediate yellow; Tuscany, sea blue; Emilia Romagna, dark red; Apulia, light purple; Piedmont, very light yellow; Calabria, black; Basilicata, light green; Valle d’Aosta, green water; autonomous province (AP) of Bolzano, fuchsia). Lineages are indicated in the figure.

The “Pangolin COVID-19 Lineage Assigner” assigned 33 to B.1 (33/58, 57%), 20 to B.1.1 (20/58, 34.4%), 1 to B.1.1.323 (1/58, 1.7%), 1 to B.1.1.61 (1/58, 1.7%), 1 to B.1.1.70 (1/58, 1.7%), and 2 genomes to lineage B (2/58, 3.5%). Figure 1 shows the lineage B.1 and descendant lineages with intermixing among viral populations sampled from different Italian regions.

The distribution on the Italian territory of the regions and autonomous provinces, indicated with the same colors reported in Figure 1, is shown in Supplementary Table S1.

A main supported clade, corresponding to lineage B.1, and a sub-clade were highlighted in the upper part of the tree. Observing the tree topology, the genomes comprised in lineage B.1 are divided in several clusters (Table 1): two genomes located in the main clade (Id. 219/2020_EPI_ISL_856884—autonomous province of Trento, northern Italy, and Id. 2591/2020_EPI_ISL_856895—Marche, center of Italy) appear phylogenetically divergent from all the others.

The isolate from Lombardy (Codogno, northern Italy, EPI_ISL_ 412973) is placed in the supported sub-clade (Figure 1, highlighted by an arrow) along with other genomes from Lombardy (EPI_ISL _451309, EPI_ISL_451308, EPI_ISL_451306), from Friuli-Venezia Giulia, northern Italy (EPI_ISL _ 417421, EPI_ISL 417423, EPI_ISL 417418), from Marche (Id. 2330/2020_EPI_ISL_855552), from Emilia-Romagna, center of Italy (206/2020_EPI_ISL_457699), from autonomous province of Trento (northern Italy) (2629/2020_EPI_ISL_457700), and also from other Italian regions, interspersed in many supported clusters within the sub-clade (Figure 1, labeled from c to i). The maximum likelihood phylogenetic tree also showed, in the lower part of the tree, a supported cluster corresponding to the lineage B (according to the nomenclature by pangolin lineage version 2021-10-13—v. 3.1.14), including seven sequences from Lazio, center of Italy (comprising the genomes of the Chinese tourist couple hospitalized at INMI), along with a genome (Id. 2249/2020_EPI_ISL_856893) collected from Friuli-Venezia Giulia (northern Italy).

### 3.2. Mutations and Glycosylation Pattern

Table 2 shows the 66 identified non-synonymous amino acid mutations.

The most frequent mutations were the following (Table 2): P323L (*nsp12*), D614G (*spike*), R203K (*nucleocapsid*), G204R (*nucleocapsid*), D3G (*membrane*), L37F (*nsp6*), G251V (*ORF3a*), V246I (*nucleocapsid)*, T175M (*membrane*), L5F (*spike*), and G50N (*ORF14*). Eight genomes belonging to lineage B, seven of which were from Lazio (central Italy) and one from the Friuli-Venezia Giulia region (northern Italy), and collected in the time period 29 January–1 March 2020, did not show the mutation D614G.

When considering the mutations occurring only among the 58 Italian genomes sequenced here, we identified additional mutations at a frequency higher than 3% (A302V in *nsp2*, D218E in *nsp3*, A81V in *nsp15*, Q218R in *nsp16*, L41F in *ORF3a*, H182R in *ORF3a*) and others at a frequency lower than 3%, as reported in Table 2.

The mutations occurring at frequencies lower than 2% in the whole Italian dataset (111 SARS-CoV-2 genomes) are also reported in Table 2.

The mutations R203K and G204R in the *nucleocapsid protein* were always detected as combined.

None of our genomes harbored the *spike protein* mutations S477N or A222V, but one genome (Id. 1177/2020_ EPI_ISL_856898 from Piedmont) presented the mutation A222S. Another mutation in the *spike*, the P681S, was identified in only one isolate (Id. 3627/2020_ EPI_ISL_856873 from Lazio).

Moving on to the glycosylation pattern, a total of 22 predicted *N*-glycosylation positions were found in SARS-CoV-2 surface glycoprotein Italian genomes (*n* = 111) by using N-GlycoSite. The positions, number, and fraction of the predicted *N*-glycosylation sites in the alignment of SARS-CoV-2 surface glycoprotein are reported in Supplementary Figure S1.

The predicted *O*-glycosylation sites for SARS-CoV-2 surface glycoprotein (111 Italian genomes) indicated sites 673 (serine), 678 (threonine), and 686 (serine) predicted as glycosylated in all the genomes, except one (Id. 3627) which showed only the site 673 (serine) predicted as glycosylated with a score of 0.5.

### 3.3. SARS-CoV-2 Gene Flows in Italy

**Figure 2:**
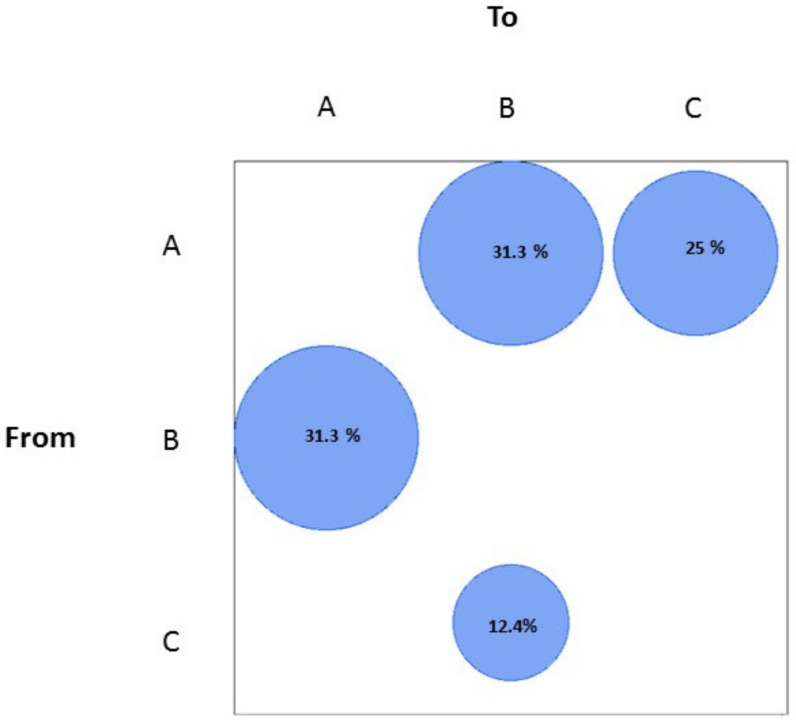
Maximum parsimony migration patterns of SARS-CoV-2 Italian genomes to/from different areas of the country (A, north; B, center; C, south). The bubblegram shows the frequency of gene flow (migrations) to/from different areas, as the percentage of total observed migrations estimated from the tree with a modified version of the Slatkin and Maddison test. Only statistically supported gene flows were reported. The surface of each circle is proportional to the percentage of observed migrations stated within the circle.

The gene flow analysis, performed according to the geographic areas north, center, and south of Italy, showed that most of the gene flow was from the north to the center (31.3%) and to the south of Italy (also including Sardinia and Sicily Islands) (25%) (Figure 2).

From the center to the north, 31.3% of gene flow was also observed. Meanwhile, a low percentage of gene flow was found from the south to the center of Italy (12.4%) (Figure 2).

**Figure 3:**
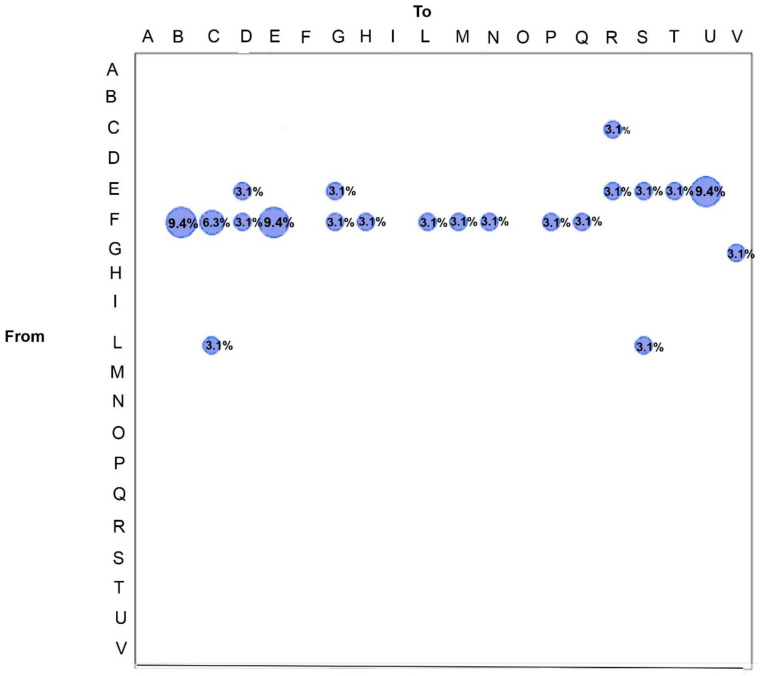
Maximum parsimony migration patterns of SARS-CoV-2 Italian genomes to/from different regions of the country (A, Basilicata; B, Friuli-Venezia Giulia; C, Lazio; D, Molise; E, Abruzzo; F, Lombardy; G, Veneto; H, Tuscany; I, Sicily; L, Campania; M, Marche; N, AP of Trento; O, Emilia Romagna; P, Apulia; Q, Piedmont; R, Umbria; S, Calabria; T, Sardinia; U, Valle d’Aosta; V, AP of Bolzano). The bubblegram shows the frequency of gene flow (migrations) to/from different regions, as the percentage of the total observed migrations estimated from the tree with a modified version of the Slatkin and Maddison test. The surface of each circle is proportional to the percentage of observed migrations stated within the circle.

Figure 3 shows the gene flow analysis conducted assigning the SARS-CoV-2 sequences according to the different Italian regions, using a modified version of the Slatkin and Maddison test in which the number of migration events required by a tree may be a good statistic to measure gene flow. Such analysis confirmed that the majority of the gene outflows occurred from Lombardy region to others. In detail, 9.4% of gene flow was observed from Lombardy to Friuli-Venezia Giulia, 6.3% from Lombardy to Lazio, 3.1% from Lombardy to Molise, 9.4% from Lombardy to Abruzzo, 3.1% from Lombardy to Veneto, and 3.1% of gene flow occurred from Lombardy to Tuscany, to Campania, to Marche, to the AP of Trento, to Apulia, to Piedmont (Figure 3).

Additionally, the Abruzzo region showed gene flow events towards Molise (3.1%), Veneto (3.1%), Umbria (3.1%), Calabria (3.1%), Sardinia (3.1%), and Valle d’Aosta (9.4%).

Other gene flows were found from Lazio to Umbria (3.1%), from Veneto to AP of Bolzano (3.1%), from Campania to Lazio (3.1%), and from Campania to Calabria (3.1%) (Figure 3).

### 3.4. Evolutionary Rate Estimate and Time-Scaled Phylogeny

Root-to-tip regression analysis of the temporal signal revealed a correlation coefficient of 0.68 and a coefficient of determination (R2) of 0.47, indicating a positive correlation and association between genetic divergence and sampling time, and attesting to the suitability of the dataset for phylogenetic molecular clock analysis.

Comparison by the BF test of the marginal likelihoods obtained by path sampling (PS) and stepping stone sampling (SS) of the strict vs. relaxed uncorrelated log-normal molecular clock showed that the second was the most appropriate for our data (taking the difference in log space, we obtained a log BF > 6 in favor of the relaxed clock).

Comparison of the different demographic models showed that the BSP is favored with respect to the constant model (log BF > 18). The BSP and the exponential growth models both best-fit the data (we obtained a log BF of 0.51 (SS) and 0.81 (PS), therefore, the difference in performance between the two models is not worth mentioning). The BSP model was confirmed as the most appropriate, as its estimates were consistent. The mean evolutionary rate estimated was 8.731 × 10^−4^ subs/site/year (95% highest posterior density (HPD) intervals 5.809 × 10^−4^ to 1.1936 × 10^−3^).

**Figure 4:**
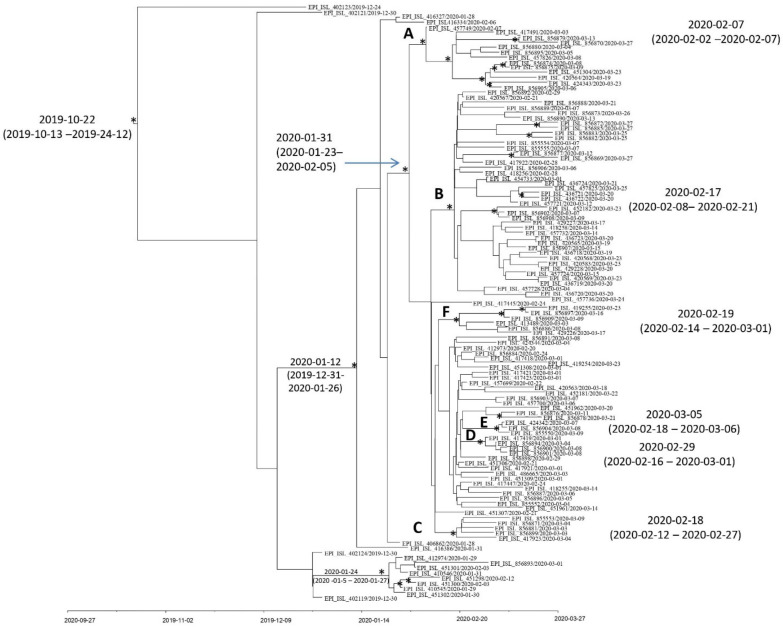
Bayesian maximum clade credibility tree representing the timescale phylogeny and date estimates for the Italian genomes. The asterisk (*) along the branches represents significant statistical support for the clade subtending that branch (posterior probability > 0.80). The scale at the bottom of the tree represents time in dates (year, month, day). Main supported clades are indicated and dated.

The Bayesian time-scaled phylogeny (Figure 4) indicated that the root of the tree dated back to 22 October 2019 (95% HPD: 13 October 2019–24 December 2020).

The mean tMRCA for the next supported internal node (also including the three Chinese isolates from Shanghai and one German genome) was estimated as 12 January 2020 (95% HPD: 31 December 2019–26 January 2020).

A large, highly supported (posterior probability, pp = 0.9987) clade (corresponding to lineage B.1 and descendent sub-lineages in Italy), including all the other Italian sequences, was identified. It dated back to the end of January (31 January 2020—95% HPD: 23 January 2019–5 February 2020). In particular, inside this clade, six statistically supported clusters (A, B, C, D, E, and F) can be highlighted (Figure 4).

Cluster A (pp = 0.96) dated back to the first days of February 2020 (95% HPD: 2–7 February 2020), and included two SARS-CoV-2 isolates from Marche (Id. 2302/2020_ EPI_ISL_856880 and 2591/2020_ EPI_ISL_856895), two from Lombardy (2561/2020 EPI_ISL_856879 and 1245/2020_EPI_ISL 457826), one from Umbria (Id. 3857/2020_EPI_ISL_856905), one from Campania (Id. 5289/2020_EPI_ISL_856870), one from Abruzzo (484/2020_EPI_ ISL_ 457749), and two from Lazio (Id. 1565/2020_ EPI_ISL_856875 and 1254/2020_EPI_ISL_856874), along with SARS-CoV-2 genomes obtained from other centers (from the GISAID database), one of which was from Abruzzo (EPI_ ISL_ 420564), two from Lazio (EPI_ ISL_ 424343 and EPI_ ISL_451304), and one from Marche (EPI_ ISL_417491). Cluster B (pp = 0.73) originated in the middle of February 2020 (95% HPD: 8–21 February 2020), and included 42 genomes sampled from the north, center, and south of Italy. Within this cluster, five statistically supported sub-clusters were found. The first of them (pp= 1) included one isolate from Tuscany (Id. 5342/2020_EPI_ISL_856885) along with one from Friuli-Venezia Giulia (Id. 5339/2020_EPI_ISL_856872), the second included two isolates from Sardinia (Id. 4608/2020_ EPI_ISL_856882 and 4618/2020_EPI_ISL_856883), the third (pp = 1) included one isolate from Campania (Id. 5287/2020_ EPI_ISL_856869) along with one from Lazio (Id 2253/2020 EPI_ISL_856877), the fourth was composed of two sequences from Abruzzo (from GISAID, EPI_ ISL_436722 and 436721), and the fifth included two genomes from Veneto (Id. 4113/2020 EPI_ISL_856908 and EPI_ISL 452182) related with one from autonomous province of Bolzano (Id. 3201/2020_EPI_ISL_856902). Cluster C (pp = 0.99) dated back to 18 February 2020 (95% HPD: 12–27 February 2020), and included one isolate from Campania (Id. 2369/2020_EPI_ISL_856871), one from Molise (Id. 1408/2020 EPI_ISL_856881), one from Lombardy (Id. 4534/2020_EPI_ISL_855553), one from Piedmont (Id. 1461/2020 EPI_ISL_856899), and one from Lazio (Id. EPI_ISL 417923). Cluster D, which included two isolates from Friuli-Venezia Giulia (Id. EPI_ISL_417419, Id. 4234/2020_EPI_ISL_856894) and two from Sicily (Id. 3779/2020 EPI_ISL_856900 and 3784/2020 EPI_ISL_856901), closely related between them (pp = 0.99), dated back to 29 February 2020 (95% HPD: 16 February–1 March 2020). Cluster E, dated to 5 March 2020 (95% HPD: 18 February–6 March 2020), included one isolate from Tuscany (Id. 4096/2020 EPI_ISL_856904), one from Lombardy (Id. 4544/2020 EPI_ISL_855550), and one genome from Lazio (EPI_ISL_424342). Cluster F, which dated back to 19 February 2020 (95% HPD: 14 February–1 March 2020), included one isolate from Veneto (Id. 4200/2020_EPI_ISL_856909), one from Molise (Id. 4926/2020 EPI_ISL_856897), two sequences from Abruzzo (Id. EPI_ISL 429226 and 3851/2020 EPI_ISL_856886), one from Lazio (Id. EPI_ISL_419255), and one from Lombardy (EPI_ISL. 413489). One statistically supported cluster corresponding to lineage B (posterior probability, pp = 1) was also highlighted (Figure 4). This cluster dated back to 24 January 2020 (95% HPD: 5–27 January 2020) and included the seven sequences from Lazio, among which were the genomes from the Chinese tourist couple hospitalized at INMI along with one genome from Friuli-Venezia Giulia (Id. 2249).

## 4. Discussion

In this study, the majority of the genomes belonged to lineage B.1, according to the classification established by Rambaut et al. [16,17]. These results are in agreement with those of other authors [6,33,34] and with data showing lineage B.1 most commonly spreading in the UK, USA, and to a lesser extent in Turkey, France, and Canada [16].

We also highlighted the presence of a relevant proportion of B.1.1 (European lineage) and a low proportion of B.1.1.323 (Northern European lineage, as reported by https://cov-lineages.org/lineages/lineageB.1.html accessed on 27 January 2022), B.1.1.61 (USA lineage), B.1.1.70, and B (lineage also found in UK, USA, China, Spain, Singapore), suggesting a considerable heterogeneity among virus strains in the country and the simultaneous co-circulation of several lineages.

We reported the first identification of B.1.1.323 lineage in Italy, in Veneto (since on the GISAID database only one B.1.1.323 sequence is available in Italy—last access 27 January 2022).

The lineage B.1.1.61 was first identified in Lombardy, then in Emilia Romagna and Umbria based on data available in GISAID (last access 27 January 2022), highlighting the circulation of this sub-lineage both in the north and in central Italy.

The B.1.1.70 was represented in a greater number of Italian regions (i.e., Friuli-Venezia Giulia, Campania, Lazio, Veneto, Umbria), with a distribution in the north and center.

SARS-CoV-2 Italian genealogy did not show compartmentalization among viral strains originating in different regions. This suggests that, before the national lockdown starting from 11 March 2020, the various lineages spread homogeneously among the regions.

Some mutations were identified at higher frequency, such as P323L (*nsp12*), D614G (*spike*), R203K (*nucleocapsid*), G204R (*nucleocapsid*), D3G (*membrane*), L37F (nsp6), G251V (*ORF3a*), V246I (*nucleocapsid*), T175M (*membrane*), and L5F (*spike*).

In particular, the first four mutations were identified at high frequency, suggesting that they could confer an evolutionary advantage to the virus. The mutation at position P323L in the RNA-dependent-RNA polymerase (*nsp12*), previously reported to be subjected to positive selection [35], may contribute to compromise the proofreading capacity by causing an increase in the mutation rate [36], or may interfere in the binding of some drugs, determining the so-called “drug resistance” [37]. The mutation D614G in the *spike protein* is an example of a mutation that became fully predominant and nearly reached fixation on a global scale [38,39]. This mutation has been the object of several hypotheses regarding its ability to confer a fitness advantage, greater infectivity, and a probable greater transmissibility, with potential impact on the severity of the disease [35]. Eight genomes belonging to lineage B, seven of which were from Lazio (central Italy) and one from Friuli-Venezia Giulia region (northern Italy), did not show the mutation D614G and were related in a supported cluster dating back to 24 January 2020 (95% HPD: 5–27 January 2020). This result is in line with the circulation in that period of a viral lineage not carrying the D614G (*spike*) and with data reported by other authors [8].

The mutations R203K and G204R in the *nucleocapsid* phosphoprotein were found in about 37% of the Italian genomes of our dataset. Some authors reported that these changes could stabilize the N structure [40].

The presence of the mutation L37F (*nsp6*) in only 7% of our isolates is not surprising, given that this change destabilizes the structure of the *nsp6* protein [38], compromising its function and possibly resulting in a relatively weak SARS-CoV-2 variant [41], or the identification of a low percentage (3.4%) of our Italian genomes showing G251V (*ORF3a*), given that this variation induces a decrease of the binding affinity of ORF3a–M and ORF3a–S complexes and affected virus assembly and transmission [42].

We reported the mutation V246I (*nucleocapsid protein*) at a higher frequency (3.6%) with respect to previous Italian studies (2.2%) [34,43]. Among the mutations identified in our 58 isolates, 12 of them (R203K, G204R, V246I, D22Y, T24N, R185H, L331F, and A381V in the *nucleocapsid protein*, G251V and H182R in ORF3a, A457V in nsp4, and P681S in the *spike protein*) fall within the B cell epitopes reported by Forni et al. [44]. Four mutations in our fifty-eight isolates (T175M in *membrane protein*, A222S in the *spike protein*, D22Y and T24N in *nucleocapsid protein*) fall within the peptides containing T cell epitopes, as reported by Peng et al. [45], indicating some variable sites, especially in the most immunogenic proteins (i.e., S, N, ORF3a). These findings could be important for planning future vaccine designs, also directed to non-*spike proteins* such as nucleocapsid, M, and ORFs [45].

In this study, we were able to highlight some mutations (T237I in *nsp3*, D2Y in *ORF3a*, P80S in nsp9, P681S in the *spike protein*, G6V in the membrane, and T24N, R185H, and A381V in the *nucleocapsid protein*) as present only in Italian genomes produced by our team, with respect to other genomes from Italy available in GISAID as of 23 February 2021 (GISAID, panel substitutions, last access 23 February 2021). Some of the above-reported changes regarded amino acids sharing similar characteristics; meanwhile, others may cause a different effect (i.e., from threonine (polar) to isoleucine (non-polar) in nsp3, and from proline (non-polar) to serine (polar) in the spike)). Regarding the *nucleocapsid protein*, the mutation R185H was located in primer binding sites used for reverse-transcription polymerase chain reaction detection assays and may have significant implications for accurate testing (https://primerscan.ecdc.europa.eu/?assay=Overview accessed on 4 December 2020).

In particular, the mutation P681S in the *spike protein* was reported as a possible immune escape role [46]. A probable first appearance of this mutation involved two genomes obtained in two regions: Lazio (lineage B.1.1, EPI_ISL_856873, collection date: 21 March 2020) and Abruzzo (lineage B.1.1, 420-EPI_ISL_1392696, collection date: 15 March 2021, but submitted to GISAID on 29 March 2021).

The findings of *O*-glycosylation for the sites 673, 678, and 686 in the *spike protein* (Italian genomes) are in agreement with observations previously reported from other countries [35,47]. One genome (Id. 3627/2020_EPI_ISL_856873) showed a lower number of predicted *O*-glycosylation sites, which can suggest a reduced immuno-evasion and protection of SARS-CoV-2 key residues. The function of the predicted O-linked glycans is unclear, but they could create a “mucin-like domain” that shields epitopes or key residues on the SARS-CoV-2 *spike protein*. Mucin-like domains as glycan shields involving immuno-evasion was reported for other viruses [47].

To the best of our knowledge, this is the first study of the gene flows conducted in Italy on genomes collected in this period. Consistent with epidemiological data, the Lombardy region was the epicenter during the first wave of COVID-19 in Italy [48]. Our data showed that the spread occurred mainly from the north, in particular from Lombardy, to the center and to the south of Italy. Our data helped to identify the locations (Italian regions) most involved in the gene flow events.

The mean evolutionary rate estimated here was 8.731 × 10^−4^ (95% HPD intervals: 5.809 × 10^−4^ to 1.19 × 10^−3^).

These values were comparable to those previously reported for SARS-CoV-2 [8,34] and to those estimated for SARS-CoV (0.80–2.38 × 10^−3^ nucleotide substitution per site per year) [49].

The SARS-CoV-2 mean evolutionary rate estimated here appears moderate if compared to that of Influenza A virus (1.8 to 2.3 × 10^−3^). The evolutionary rate is one of the most fundamental aspects of sequence evolution. If a virus evolves relatively slowly, there will be a better chance for development of effective long-lasting vaccines and successful treatment for patients.

The Bayesian time-scaled phylogeny showed that the date of origin of the supported Italian cluster corresponding to lineage B (according to the nomenclature by pangolin v. 2.3.0—version 21 February 2021) dated back to 24 January 2020 (95% HPD: 5–27 January 2020), in line with other results [8].

Our dated tree also suggested that SARS-CoV-2 lineage B.1 probably entered Italy between the end of January and early February 2020, and this finding is consistent with the literature [8,33,50,51,52].

Six internal statistically supported clusters (A, B, C, D, E, F) were dated back in different time periods, ranging from 7 February 2020 (cluster A, the oldest one) to 5 March 2020 (cluster E, the most recent). This may suggest that the virus continued the circulation and dissemination among the different Italian regions in addition to the local transmission.

Of course, a possible bias of the study that cannot be ruled out is the selection of SARS-CoV-2 genomes, which are represented by those available in the database, and may be not necessarily representative of the real-world situation.

In conclusion, we have provided a picture of the predominant strains circulating during the early stage of the COVID-19 pandemic in Italy. Furthermore, we performed a gene flow analysis of SARS-CoV-2 among Italian regions, allowing an improved characterization of SARS-CoV-2.

## Figures and Tables

**Table 1:** Clades and clusters, lineage assignment, number of SARS-CoV-2 genomes, and regions/autonomous provinces referred to in the maximum likelihood tree reported in Figure 1.

Clade	Cluster	Lineage	Total	Region and Number
main	a	B.1	6	Umbria (1), Lazio (4), Abruzzo (1)
main	b	B.1	2	Campania (1), Lombardy (1)
sub-clade	c	B.1	6	Veneto (1), Molise (1), Abruzzo (2), Lazio (1), Lombardy (1)
sub-clade	d	B.1	3	Lombardy (1), Lazio (1), Tuscany (1)
sub-clade	e	B.1	4	Sicily (2), Friuli-Venezia Giulia (2)
sub-clade	f	B.1	2	Marche (1), Lombardy (1)
sub-clade	g	B.1	2	Campania (1), Apulia (1)
sub-clade	h	B.1	5	Piedmont (1), Molise (1), Lombardy (1), Campania (1), Lazio (1)
sub-clade	i	B.1	2	Lazio (2)
sub-clade	l	B.1.1 (39), B.1.1.323 (1)	42	Abruzzo (16), Tuscany (2), Veneto (2), AP of Bolzano (1)
				Lazio (3), Campania (2), Calabria (1), Lombardy (3), Sardinia (3), Valle d’Aosta (3), Umbria (1), Sicily (1), Basilicata (2)
		B.1.1.61 (1)		Friuli-Venezia Giulia (1), Molise (1).
		
		B.1.1.70 (1)		

**Table 2:** The non-synonymous amino acid mutations harbored by the Italian genomes (*n* = 111) investigated here and non-synonymous amino acid mutations harbored by the 58 Italian genomes collected from the Istituto Superiore di Sanità and from the Italian Scientific Department of the Army Medical Center. The amino acids that resulted converted to a stop codon (*) are also reported.

Mutation	*n*/Total	Percentage	*n*/58 (ISS and Army Medical Center)	Percentage	Target	Gene
P323L	103/111	92.80%	56/58	96.50%	*nsp12-RNA-dependent RNA polymerase*	*ORF1ab*
D614G	103/111	92.80%	56/58	96.50%	*spike*	*S*
R203K	41/111	37%	23/58	39.70%	*nucleocapsid*	*N*
G204R	41/111	37%	23/58	39.70%	*nucleocapsid*	*N*
D3G	13/111	11.70%	9/58	15.50%	*membrane*	*M*
L37F	9/111	8.10%	4/58	6.90%	*nsp6*	*ORF1ab*
G251V	8/111	7.20%	2/58	3.44%	*ORF3a*	*ORF3a*
V246I	4/111	3.60%	3/58	5.20%	*nucleocapsid*	*N*
T175M	3/111	2.70%	2/58	3.44%	*membrane*	*M*
L5F	3/111	2.70%	2/58	3.44%	*spike*	*S*
A302V	2/111	1.80%	2/58	3.44%	*nsp2*	*ORF1ab*
D218E	2/111	1.80%	2/58	3.44%	*nsp3*	*ORF1ab*
S74A	2/111	1.80%	1/58	1.72%	*nsp13/helicase*	*ORF1ab*
A81V	2/111	1.80%	2/58	3.44%	*nsp 15/endoRNAse*	*ORF1ab*
Q218R	2/111	1.80%	2/58	3.44%	*nsp16/2’-O-ribose methyltransferase*	*ORF1ab*
L41F	2/111	1.80%	2/58	3.44%	*ORF3a*	*ORF3a*
H182R	2/111	1.80%	2/58	3.44%	*ORF3a*	*ORF3a*
Q12 *	2/111	1.80%	2/58	3.44%	*ORF10*	*ORF10*
P191S	1/111	0.90%	1/58	1.72%	*nsp2*	*ORF1ab*
T237I	1/111	0.90%	1/58	1.72%	*nsp3*	*ORF1ab*
A496V	1/111	0.90%	1/58	1.72%	*nsp3*	*ORF1ab*
A534V	1/111	0.90%	1/58	1.72%	*nsp3*	*ORF1ab*
P1159S	1/111	0.90%	/	/	*nsp3*	*ORF1ab*
S1285F	1/111	0.90%	/	/	*nsp3*	*ORF1ab*
I1413L	1/111	0.90%	/	/	*nsp3*	*ORF1ab*
M1547I	1/111	0.90%	/	/	*nsp3*	*ORF1ab*
N1785D	1/111	0.90%	1/58	1.72%	*nsp3*	*ORF1ab*
A1872V	1/111	0.90%	/	/	*nsp3*	*ORF1ab*
V357I	1/111	0.90%	1/58	1.72%	*nsp4*	*ORF1ab*
A457V	1/111	0.90%	1/58	1.72%	*nsp4*	*ORF1ab*
K285N	1/111	0.90%	/	/	*nsp6*	*ORF1ab*
A287S	1/111	0.90%	/	/	*nsp6*	*ORF1ab*
S10L	1/111	0.90%	/	/	*nsp7*	*ORF1ab*
F49I	1/111	0.90%	/	/	*nsp7*	*ORF1ab*
Y149 *	1/111	0.90%	/	/	*nsp8*	*ORF1ab*
W154R	1/111	0.90%	/	/	*nsp8*	*ORF1ab*
P80S	1/111	0.90%	1/58	1.72%	*nsp9*	*ORF1ab*
R285H	1/111	0.90%	1/58	1.72%	*nsp12-RNA-dependent RNA polymerase*	*ORF1ab*
S363R	1/111	0.90%	1/58	1.72%	*nsp12-RNA-dependent RNA polymerase*	*ORF1ab*
D893Y	1/111	0.90%	/	/	*nsp12-RNA-dependent RNA polymerase*	*ORF1ab*
P529L	1/111	0.90%	1/58	1.72%	*nsp13/helicase*	*ORF1ab*
T31I	1/111	0.90%	1/58	1.72%	*nsp14/3’-to-5’ exonuclease*	*ORF1ab*
V287F	1/111	0.90%	1/58	1.72%	*nsp14/3’-to-5’ exonuclease*	*ORF1ab*
P23S	1/111	0.90%	/	/	*nsp 15/endoRNAse*	*ORF1ab*
S154F	1/111	0.90%	/	/	*nsp 15/endoRNAse*	*ORF1ab*
K160R	1/111	0.90%	1/58	1.72%	*nsp16/2’-O-ribose methyltransferase*	*ORF1ab*
A222S	1/111	0.90%	1/58	1.72%	*spike*	*S*
P681S	1/111	0.90%	1/58	1.72%	*spike*	*S*
D839Y	1/111	0.90%	/	/	*spike*	*S*
M1T	1/111	0.90%	1/58	1.72%	*ORF3a*	*ORF3a*
D2Y	1/111	0.90%	1/58	1.72%	*ORF3a*	*ORF3a*
Q57H	1/111	0.90%	1/58	1.72%	*ORF3a*	*ORF3a*
A110S	1/111	0.90%	/	/	*ORF3a*	*ORF3a*
G6V	1/111	0.90%	1/58	1.72%	*membrane*	*M*
W29R	1/111	0.90%	1/58	1.72%	*ORF7b*	*ORF7b*
D22Y	1/111	0.90%	1/58	1.72%	*nucleocapsid*	*N*
T24N	1/111	0.90%	1/58	1.72%	*nucleocapsid*	*N*
D103Y	1/111	0.90%	/	/	*nucleocapsid*	*N*
R185H	1/111	0.90%	1/58	1.72%	*nucleocapsid*	*N*
L331F	1/111	0.90%	1/58	1.72%	*nucleocapsid*	*N*
A381V	1/111	0.90%	1/58	1.72%	*nucleocapsid*	*N*
G50N	41/111	37%	23/58	39.7%	*ORF14 (uncharacterized protein)*	*ORF14*
G50E	1/111	0.90%	1/58	1.72%	*ORF14 (uncharacterized protein)*	*ORF14*
V32I	1/111	0.90%	1/58	1.72%	*ORF14 (uncharacterized protein)*	*ORF14*
R32L	1/111	0.90%	1/58	1.72%	*ORF9b*	*ORF9b*
L21M	1/111	0.90%	1/58	1.72%	*ORF9b*	*ORF9b*
Q18H	1/111	0.90%	1/58	1.72%	*ORF9b*	*ORF9b*
G71S	1/111	0.90%	1/58	1.72%	*nsp5*	*ORF1ab*

## Data Availability

Data are contained within the article and figures. All the sequences are available in the GISAID database.

## Supplementary Materials

The following supporting information can be downloaded at: https://www.mdpi.com/article/10.3390/v14030472/s1, Figure S1. (a) The predicted *N*-glycosylation sites in SARS-CoV-2 *surface* glycoprotein Italian genomes obtained by using N-GlycoSite tool. (b) The positions, number and fraction of the predicted *N*-glycosylation sites. Table S1. The table reported the localization on the Italian territory of the regions/autonomous provinces that have been indicated with the same colors reported in the Maximum Likelihood Phylogenetic tree. Abruzzo, blue; Lazio, red; Lombardy, green; Friuli-Venezia Giulia, pink-fuchsia; Marche, grey; Veneto, light blue; Molise, violet; Sicily, ocra yellow; Sardinia, pink flesh; Campania, dark green; autonomous province (AP) of Trento, dark grey; Umbria, intermediate yellow; Tuscany, sea blue; Emilia Romagna, dark red; Apulia, light purple; Piedmont, very light yellow; Calabria, black; Basilicata, light green; Valle d’Aosta, green water; autonomous province (AP) of Bolzano, fuchsia.
