# Supplementary material for: Analysis of Genomic Characteristics of SARS-CoV-2 in Italy, 29 January to 27 March 2020
Source: Viruses. 2022 Feb 25;14(3):472. doi: 10.3390/v14030472 (PMC8951147; doi:10.3390/v14030472)
Supplement: Supplementary file 1 [file viruses-14-00472-s001.zip › viruses-1300487-supplementary.pdf]

viruses

MDPI

Supplementary Materials

Analysis of Genomic Characteristics of SARS-CoV-2 in Italy, 29 January to 27 March 2020

a)

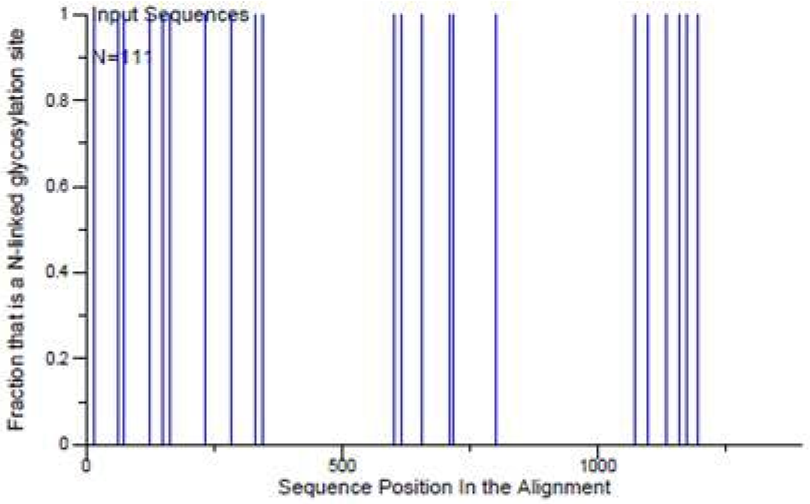

b)

Sequence position(s) of High N-glycosylation Site (>60%)

Pos	Top seq	Num of N-glycosylation	Fraction
17	N	111	1.000
61	N	111	1.000
74	N	111	1.000
122	N	111	1.000
149	N	111	1.000
165	N	111	1.000
234	N	111	1.000
282	N	111	1.000
331	N	111	1.000
343	N	111	1.000
603	N	111	1.000
616	N	111	1.000
657	N	111	1.000
709	N	111	1.000
717	N	111	1.000
801	N	111	1.000
1074	N	111	1.000
1098	N	111	1.000
1134	N	111	1.000
1158	N	111	1.000
1173	N	111	1.000
1194	N	111	1.000

Figure S1. (**a**) The predicted N-glycosylation sites in SARS-CoV-2 surface glycoprotein Italian genomes obtained by using N-GlycoSite tool. (**b**) The positions, number and fraction of the predicted N-glycosylation sites were reported.

Table S1. The table reported the localization on the Italian territory of the regions / autonomous provinces that have been indicated with the same colors reported in the Maximum Likelihood Phylogenetic tree (Figure 1). Abruzzo, blue; Lazio, red; Lombardy, green; Friuli-Venezia Giulia, pink-fuchsia; Marche, grey; Veneto, light blue; Molise, violet; Sicily, ocra yellow; Sardinia, pink flesh; Campania, dark green; autonomous province (AP) of Trento, dark grey; Umbria, intermediate yellow; Tuscany, sea blue; Emilia Romagna, dark red; Apulia, light purple; Piedmont, very light yellow; Calabria, black; Basilicata, light green; Valle d'Aosta, green water; autonomous province (AP) of Bolzano, fuchsia.

Regions and Autonomous Provinces (Italy)	localization
Valle d' Aosta	North
Piedmont	North
Lombardy	North
Autonomous province of Bolzano	North
Autonomous province of Trento	North
Friuli – Venezia - Giulia	North
Veneto	North
Emilia - Romagna	North
Tuscany	Center
Marche	Center
Umbria	Center
Lazio	Center
Abruzzo	Center
Molise	Center
Campania	South
Apulia	South
Basilicata	South
Calabria	South
Sicily	South
Sardinia	South
